# Theoretical Studies of Cyanophycin Dipeptides as Inhibitors of Tyrosinases

**DOI:** 10.3390/ijms23063335

**Published:** 2022-03-19

**Authors:** Agnieszka Krzemińska, Natalia Kwiatos, Franciela Arenhart Soares, Alexander Steinbüchel

**Affiliations:** International Center for Research on Innovative Biobased Materials (ICRI-BioM)—International Research Agenda, Lodz University of Technology, Żeromskiego 116, 90-924 Lodz, Poland; agnieszka.krzeminska-kowalska@p.lodz.pl (A.K.); franciela.arenhart-soares@p.lodz.pl (F.A.S.); alexander.steinbuchel@p.lodz.pl (A.S.)

**Keywords:** tyrosinase, molecular docking, AlphaFold, tyrosinase-related protein 1, tyrosinase-related protein 2

## Abstract

The three-dimensional structure of tyrosinase has been crystallized from many species but not from Homo sapiens. Tyrosinase is a key enzyme in melanin biosynthesis, being an important target for melanoma and skin-whitening cosmetics. Several studies employed the structure of tyrosinase from *Agaricus bisporus* as a model enzyme. Recently, 98% of human genome proteins were elucidated by AlphaFold. Herein, the AlphaFold structure of human tyrosinase and the previous model were compared. Moreover, tyrosinase-related proteins 1 and 2 were included, along with inhibition studies employing kojic and cinnamic acids. Peptides are widely studied for their inhibitory activity of skin-related enzymes. Cyanophycin is an amino acid polymer produced by cyanobacteria and is built of aspartic acid and arginine; arginine can be also replaced by other amino acids. A new set of cyanophycin-derived dipeptides was evaluated as potential inhibitors. Aspartate–glutamate showed the strongest interaction and was chosen as a leading compound for future studies.

## 1. Introduction

Melanin is a high molecular weight biopolymer derived from tyrosine and plays a key role in the natural UV protection of skin [1,2,3]. Besides light absorption, melanin can act as a radical scavenger and presents metal chelating and redox properties [4]. However, dysfunctions in melanin biosynthesis at melanocytes can lead to hyperpigmentation causing either aesthetical problems or melanoma, one of the most aggressive forms of skin cancers [3,5,6,7]. It is estimated that melanoma causes globally approximately 55,000 deaths per year, mainly due to the multiple resistance to the currently available anticancer agents [8]. On the other hand, the lack of produced melanin in humans and animals results in a condition known as oculocutaneous albinism [9,10]. Recently, epidemiological evidence was found relating melanoma and Parkinson’s disease, a neurodegenerative disorder associated with a loss of dopaminergic neurons in the brain [11,12].

**Figure 1:**
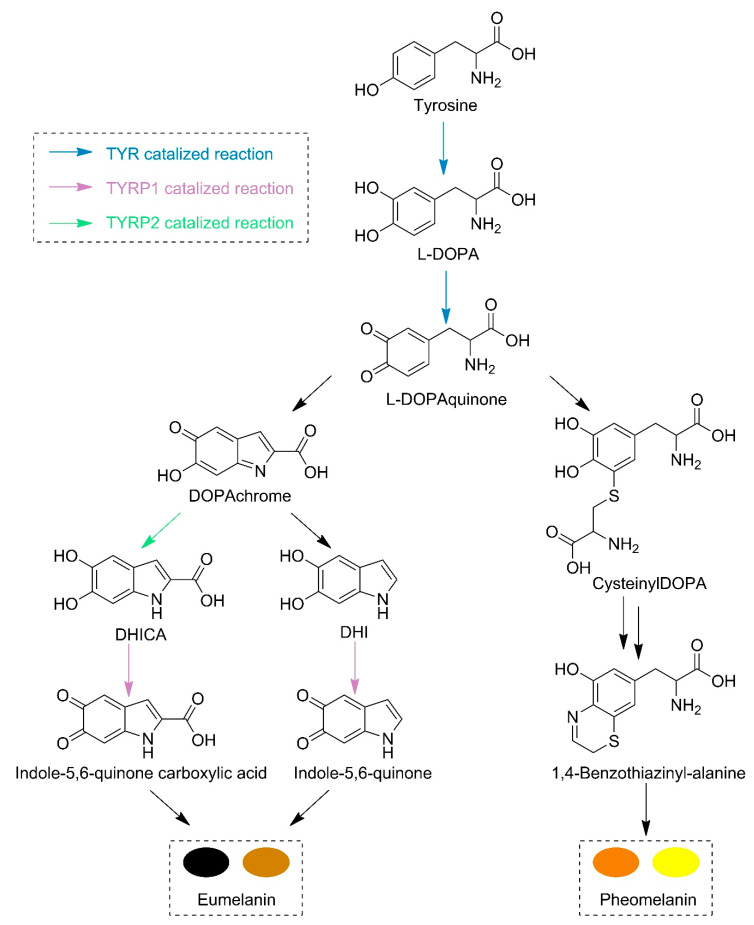
Proposed melanin biosynthesis involving the three tyrosinase-related proteins.

Although the biosynthesis of melanin is complex and involves several steps, so far only three main tyrosine-like proteins have been identified: tyrosinase (TYR), tyrosinase-related protein 1 (TYRP1), and tyrosinase-related protein 2 (TYRP2) [13]. Tyrosinase (a monophenol monooxygenase, EC 1.14.18.1) is a metalloprotein possessing two copper atoms in the active site; it is considered the rate-limiting enzyme of melanogenesis [14]. TYR is present in the whole body and possesses several biological roles [10]. Chemically, TYR catalyzes two main reactions (Figure 1). The first one is related to the hydroxylation of tyrosine to form L-3,4-dihydroxyphenylalanine (L-DOPA). The second reaction involves the oxidation of the hydroxyl groups into ketones (DOPAquinone). TYRP1 (E.C. 5.3.3.12), on the other hand, possesses two zinc atoms in the active site and is a melanocyte-specific protein. Although in mice TYRP1 catalyzes the oxidation of 5,6-dihydroxy-indole-2-carboxylic acid, this reaction was not confirmed in humans [13]. While TYRP1 has a structural role, contributing to the stabilization of the melanocyte, TYRP2 is a tautomerase (EC 5.3.2.3) and catalyzes the conversion of DOPAchrome into 5,6-dihydroxyindole-2-carboxylic acid (DHICA) [15]. It is also assumed that TYRP2 plays an important role in the cytoprotection of melanocytes [16].

Since melanins have many different roles in the human body, numerous studies were conducted employing tyrosinase as a target for inhibitor compounds [17,18,19,20,21]. Molecules such as 4-hydroxy-cinnamic acid (CA) and kojic acid (KA) have recently received much attention due to their ability to bind to tyrosinase (IC_50CA_ = 0.50 mM and IC_50KA_= 30.6 μM, respectively) [20,22,23]. Due to the high structural similarity between CA and the TYR substrate, tyrosine, and since KA was one of the first inhibitors identified, both structures are commonly used as a reference for docking studies [24,25]. However, recently, Roulier et al. pointed out that KA can act as an inhibitor only for mushroom tyrosinase, but is only a weak inhibitor for the human enzyme. In addition, due to the nonspecific nature of tyrosinase for substrate, molecules such as CA can act as substrates, not real inhibitors [8].

Cyanophycin, or multi-L-arginyl-poly-L-aspartate, is a polymer consisting of amino acids; it is produced by cyanobacteria and also some heterotrophic bacteria. It is composed of aspartic acid in the backbone and arginine residues as the side chains [26]. However, its composition is variable and depends on the conditions of polymerization and the specificity of cyanophycin synthetase responsible for catalysis of the polymerization reaction. Among others, the following amino acids were found in the side chain of cyanophycin: lysine, glutamic acid, citrulline, canavanine, homoarginine, ornithine, and β-methyl ester [27,28,29,30]. Although the application of this biopolymer is not well established, the importance of dipeptides and peptides derived from it was already discussed [31,32]. Peptides, especially those derived from natural sources, as is the case of cyanophycin-derived peptides, are widely studied for their inhibitory activity for aging-related enzymes, involving tyrosinase [21,33,34]. Unlike KA and CA, the proposed peptides do not possess phenol groups similar to tyrosine. However, the presence of carboxylic functions and amino groups could be interesting for interactions with the metals atoms present in the catalytic site [35]. 

Although TYR is an abundant protein, the crystal structure for the human protein has not been determined yet. The studies investigating tyrosinase inhibitors often use the *Agaricus bisporus* tyrosinase (abTYR) crystal structure (Protein Data Bank, PDB ID: 2Y9X), arguing with its close relationship with the human enzyme [36,37]. However, as pointed out by Roulier et al., both enzymes have several dissimilarities, and in silico results obtained with abTYR should be considered carefully [8]. In July, the CASP14-winning AlphaFold2 algorithm was published and the AlphaFold Protein Structure Database was released [38,39]. The database contains structure models of over 98.5% of the human proteins [39], including TYR and TYRP2, providing new protein structures for in silico studies. 

In this paper, the structures of human tyrosinase, TYRP1, TYRP2, and abTYR were compared and analyzed in presence of the ligands KA and CA. This study also investigated cyanophycin-derived peptides as potential inhibitors of human tyrosinase, TYRP1, and TYRP2 enzymes. Since computational methodologies represent an important tool for the fast screening of new potential compounds with biological activity against a chosen target [40,41], e.g., to predict binding affinities, the interactions of the selected enzymes with a library of cyanophycin-derived compounds were conducted, allowing the identification of a leading candidate. Such compounds might be an important component for new drug discovery for the treatment of such diseases as cancer or Parkinson’s disease. The present study consists of key steps to the rational development of new tyrosinase inhibitors through computer-aided methodologies.

## 2. Results

The great interest in the inhibition of tyrosinases led to a plethoric literature data set, especially when targeting mushroom tyrosinase (abTYR), for which the crystal structure has been available for 10 years. In addition, the costs for experiments in the wet laboratory are for this enzyme much lower than those for human tyrosinase. For instance, in the literature, the binding affinities for KA and CA in complex with abTYR (PDB ID: 2Y9X) can be found. Binding affinities of −5.5 and −5.8 kcal∙mol^−1^ for KA and CA, respectively, have been calculated by using AutoDock Vina [37]. However, it is difficult to judge the accuracy of these values, especially when they are compared with experimental values obtained from the inhibition constant K_I_. Two different values of free energy of binding −4.6 and −7.2 kcal∙mol^−1^ were reported in the literature for CA [22,42]. Moreover, for KA, free energy of binding values vary from −2.8 to −7.3 kcal∙mol^−1^ [43,44,45]. 

Having that in mind, the calculation for new ligands should always be compared to model ligands such as KA and CA. The winning complexes would be those with lower binding affinities than those for KA and CA. KA is a natural chelating agent produced as a metabolite by several bacteria and fungi, and it is sold as a well-known cosmetic product that promotes skin whitening [20]. CA is known for its wide scope of pharmacological properties such as antioxidant, antibacterial, antifungal, and anti-inflammatory properties [46].

### 2.1. Tyrosinase Structure Comparison

In this study, TYR modeled by AlphaFold was used for the first time in docking studies. This model obtained a high predicted Local Distance Difference Test (pLDDT) score and expected position error close to zero along with the whole protein [47]. Ramachandran plot prepared by MolProbity [48] shows that 97% of the atoms are located in favored positions, while none are in outlier regions. Although the AlphaFold structures are complete, with excellent resolution and almost perfect match confirmed by Ramachandran plots, crucial stabilizing cations are missing. The model was enriched with copper atoms as explained in the methodology section. Figure S1 shows the distances between copper atoms at abTYR and TYR enzymes with distances between 2.1 and 2.3 Å in both cases. This is in good correlation with previous studies [49,50], which proves the correct structure preparation. 

The TYR 3D structure was not available until July 2021 when DeepMind released its database with a humongous number of predicted protein structures. As tyrosinase inhibition studies are important due to their impact on medicine and drug development, scientists used abTYR for this purpose. It was argued that mushroom tyrosinase is closely related to the human enzyme in terms of kinetics and probable 3D architecture [36,37]. However, it was already stated that results obtained with abTYR cannot be directly applied also to human tyrosinase [8,51]. Nevertheless, for the first time, we have here the possibility to compare the abTYR with spherically correct TYR structure. According to the structural alignment done with Chimera v1.14 [52], these structures are only 13% identical, which disqualifies usage of abTYR for human tyrosinase studies. 

**Figure 2:**
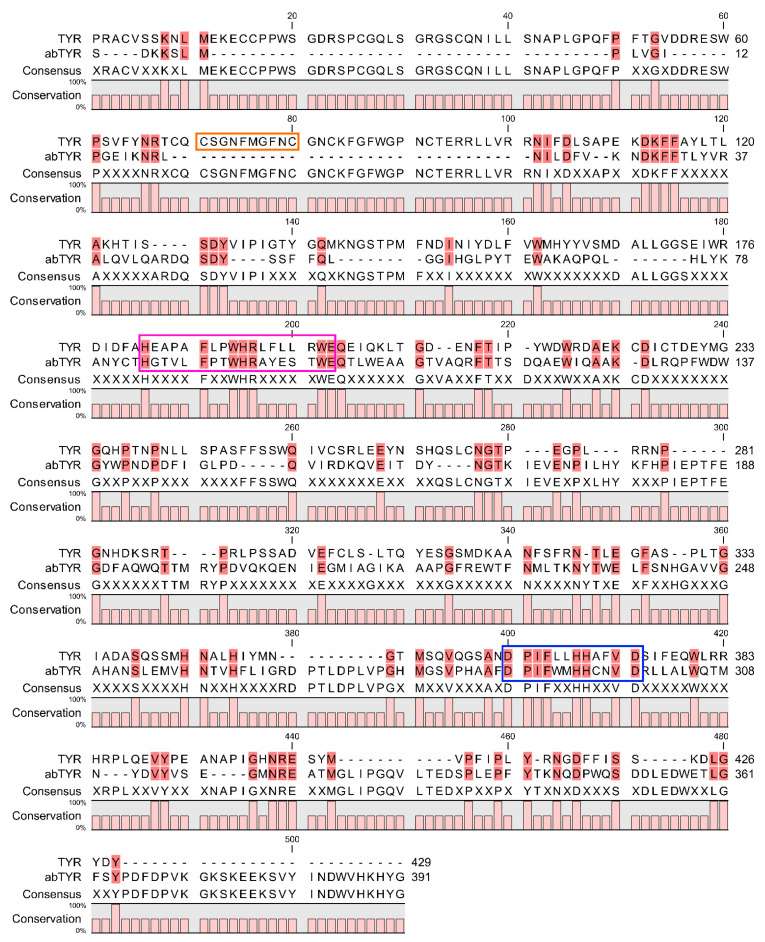
Pairwise sequence alignment of TYR and abTYR sequences used in this study. Magenta, CuA binding pattern; blue, CuB binding pattern; orange, EFG pattern.

**Figure 3:**
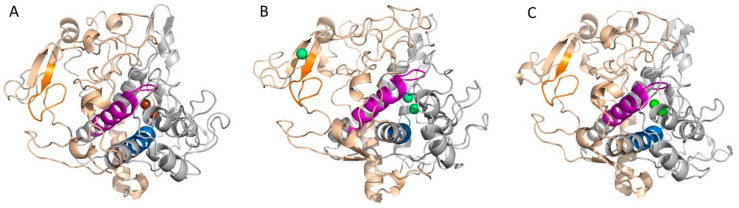
Three-dimensional structures of tyrosinases: (**A**) TYR, (**B**) TYRP1, (**C**) TYRP2. Grey, tyrosinase domain; magenta, CuA binding pattern; blue, CuB binding pattern; orange, EFG pattern; brown, copper atoms; green, zinc atoms.

The *A. bisporus* tyrosinase sequence (C7FF04) shows 24% identity to the human tyrosinase sequence (P14679) (Figure 2). AbTYR is a 576 amino acid long protein, which contains a tyrosinase domain between amino acids 51 and 309 and a tyrosinase C-terminal domain between amino acids 424 and 544. TYR has 529 amino acids with the tyrosinase domain located between amino acids 170 and 430 (Figure 2 and Figure 3). Both sequences possess the tyrosinase and hemocyanin CuB-binding region signature D-P-x-F-[LIVMFYTW]-x(2)-H-x(3)-D and the CuA-binding region signature H-x(4,5)-F-[LIVMFTP]-x-[FW]-H-R-x(2)-[LVMT]-x(3)-E (Figure 2 and Figure 3). According to a previous study, seven histidine residues (His180, His202, His211, His363, His367, His389, His390) participate in copper chelation and substrate binding [10,53]. Human tyrosinase possesses the EGF domain (Figure 2 and Figure 3), which is absent in the mushroom tyrosinase. Moreover, TYR is a membrane-bound protein that possesses seven N-glycosylation sites [8]. 

TYRP1 and TYRP2 are the enzymes responsible for the subsequent reactions in melanin formation (Figure 1). The TYRP1 crystal structure is known (5M8L) and was used in this study [13], while the TYRP2 structure was obtained from the AlphaFold Protein Structure Database. TRP1 has a sequence of 537 amino acids (P17643), while TRP2 has 519 amino acids (P40126). The three proteins are closely related to each other, and it was hypothesized that TYRP1 evolved from TYR by duplication and then mutated to TYRP2. TYR is 44% identical to TYRP1 and TYRP2, while TYRP1 is almost 55% identical to TYRP2. However, they exhibit a similar 3D structure with analogous domain localization (Figure 3, Figures S2 and S3). TYRP1 and TYRP2 possess zinc ions in the place of copper in TYR; thus, they are not able to perform redox reactions [8].

### 2.2. Molecular Docking Studies with TYR and abTYR

KA and CA are molecules often described as potent inhibitors of tyrosinase [23,46,50,54]. This computational study reports on the docking of these two molecules to the structure of human tyrosinase. KA enters the active pocket of human tyrosinase with its carbonyl group facing copper atoms and interacting with them. Moreover, Val377, His363, and His202 create π-interactions with KA, while van der Waals interactions are observed with the following residues: His180, Glu203, Phe207, Phe347, His367, Ser380, Phe386, and His390 (Figure S4). CA also interacts with copper atoms, as well as with His367 and Val377 by π-interactions. Hydrogen bonds occur with His363. Other amino acids that take part in the CA binding are His180, Ser184, Asp199, Glu203, Phe347, Asn364, His363, Ser380, Phe386, and His390.

According to previous studies and also this study, Asn260 of abTYR is responsible for the binding of the inhibitor by van der Waals interactions [49,55]. This residue corresponds to Asn364 of TYR and interacts with KA and CA in a similar manner. Val283 has been discussed before to form π–π stacking interactions with ligands [49,56], and this is also true for Val377 in TYR. Other similar interactions can be noticed for Phe90, Ala287, and Phe292 of abTYR and Phe207, Ser380, and Phe386 of TYR (Figure S5). 

Another residue of abTYR mentioned in the literature, which interacts with inhibitors by van der Waals forces, is Met280 [49,56]. This methionine residue is aligned in TYR at position 374; however, it is situated too far from docked KA or CA to be able to interact with it. Similarly, Phe264 is described to interact by van der Waals forces with a ligand [55,56], while the interaction of a ligand with the corresponding Ile368 cannot occur due to a too large distance between the atoms. Gly281, Ser282, and Thr261 of abTYR were described to be important for inhibitor binding [54]. However, their aligned residues in TYR Ser394 and Ala385 are located too far away from the docked ligands. On the other hand, we found residues interacting with inhibitors in TYR whose respective residues from abTYR could not interact with them, such as Glu203, Glu345, and Phe347.

Summing up, the active sites of abTYR and human TYR differ quite significantly, and the described differences have a huge impact on ligand binding. Moreover, the overall structure is quite distinct (RMSD 8.8 Å). The 3D structure of the whole protein may be important for its activity due to allosteric regulations or electrostatic forces. Moreover, interactions of TYR with TYRP1 and TYRP2 are important for their activity. TYR, TYRP1, and TYRP2 work as a complex, and TYRP1 and TYRP2 stabilize TYR, thus modulating its activity [57,58]. Having identified these dissimilarities, we concluded that for further research, the AlphaFold TYR structure has to be used because previously obtained models were not as accurate. For example, 92% of atoms are situated in a Ramachandran favored region in the model obtained by Swiss-Model [59] employing TYRP1 crystal structure as a template, while for the AlphaFold structure it is 97%.

Eight cyanophycin-derived peptides (Table 1) were docked to the mushroom and human tyrosinase enzymes in order to evaluate their potential as inhibitors for the tyrosinase proteins. All ligands showed similar binding affinities to the enzymes. However, Lig2—aspartate–glutamate—showed the closest binding affinity values to KA. According to Karkaya et al., the interaction of the inhibitor with Cu atoms is crucial for the inhibition [54]. Interestingly, in the case of Lig2, no interactions with Cu atoms were identified when the docking was conducted with abTYR. However, when Lig2 was docked with human TYR, metal acceptor and van der Waals interactions between the Lig2 and both Cu atoms were found. While in TYR the distance between the Lig2 and the Cu atoms is in the range of 2.5 to 3.3 Å, larger distances were observed for abTYR (5.1 to 5.6 Å), which is too far to establish good coordination with the metal atoms [13]. Additionally, hydrophobic interactions with other amino acid residues present in the binding pocket of both enzymes are slightly different, indicating distinct binding modes for Lig2. For example, for abTYR, van der Waals interactions are observed for histidine residues His259, His85, and His263. In human TYR, Lig2 relates with His180 through π–sigma interactions and with His363 through van der Waals interaction. In human TYR two additional attractive charge interactions between His367 and Arg308 were also observed. 

### 2.3. Molecular Docking Studies with TYRP1 and TYRP2

TYRP1, unlike TYR, is not a redox enzyme due to the presence of two zinc atoms instead of copper atoms in the active site [13]. In addition, TYRP1 can bind the same substrates and inhibitors as TYR. We performed docking studies with KA, CA, and the cyanophycin-derived ligands. The calculated binding affinities can be seen in Table 1. 

Similar to the results of the investigations obtained for TYR, CA has a slightly more negative binding affinity to TYRP1 than KA. Although both compounds are metal chelators, no direct interactions with the zinc atoms were found, in contrast to what Lai et al. reported for tropolone [13]. For CA, the distance of the carboxylic group to the zinc atoms is around 4.3 to 4.5 Å, which is too far to chelate the metal. Although the distances for KA to the metal atoms are slightly smaller (3.1 to 3.5 Å), still no binding to the zinc atom was found [13]. For KA, four hydrogen-bonding interactions were found in the active site of the enzyme, with Tyr362, Ser394, Gly388, and His381, while for CA van der Waals interactions were identified for Thr391. Regarding the ligands, Lig2 and Lig4 showed the lowest binding affinities with TYRP1. Interestingly, for this enzyme, Lig4 had the most favorable binding affinity. When analyzing the interactions with the enzyme in more detail, it was possible to identify several charged interactions of Lig4 with both zinc atoms and also with the residues Glu216, Asp212, and His381. This may be the reason for the lowest binding affinity, as such interactions were not visible for other ligands (Figure S6D). 

TYRP2 is also known as DOPAchrome tautomerase since it catalyzes the conversion of DOPAchrome into 5,6-dihydroxyindole-2-carboxylic acid (DHICA) (see Figure 1). It is important to mention that in the absence of TYRP2, the DOPAchrome is spontaneously converted into DHI [60]. Once DHI is produced, it cannot be chemically converted back to DHICA, and the indole-5,6-quinone is polymerized into a high molecular weight dark biopolymer [61]. Moreover, TYRP2 plays several regulative roles in melanocyte functioning, being often chosen as a target for melanoma cancer therapy. As can be seen in Table 1, all cyanophycin-derived ligands, as well KA and CA, showed similar binding affinities for both TYR and TYRP1 enzymes. Moreover, KA and CA have slightly higher binding affinities than the ligands, probably due to the presence of phenolic rings which can interact through π–sigma contacts (Figures S6 and S7). Among the ligands, again Lig2 showed binding affinity comparable to KA and CA. A hydrogen-bond interaction between Lig2 and Ser351 of TYRP2 was identified, as were interactions of the carbonyl group from glutamic acid with the zinc ions (Figure S7C). Additionally, Lig4 showed a similar binding affinity to Lig2 in the presence of TYRP2. No interactions of Lig4 with the metal atoms were found. Interactions with the metal atoms are visible for Lig2 and may be a reason for a stronger binding of Lig2. Moreover, the size and shape of Lig2 differ from those of Lig4. Lig2 is over 10 Å long and only 2.4 Å wide, whereas Lig4 is two times shorter and twice as wide as Lig2, which undoubtedly influences the interactions with the active site. The active site is 5.5 Å wide near the copper atoms and almost 8 Å wide closer to the entrance, which may be also a reason why the wider ligands do not interact with Cu atoms—they cannot fit as close as Lig2. 

**Figure 4:**
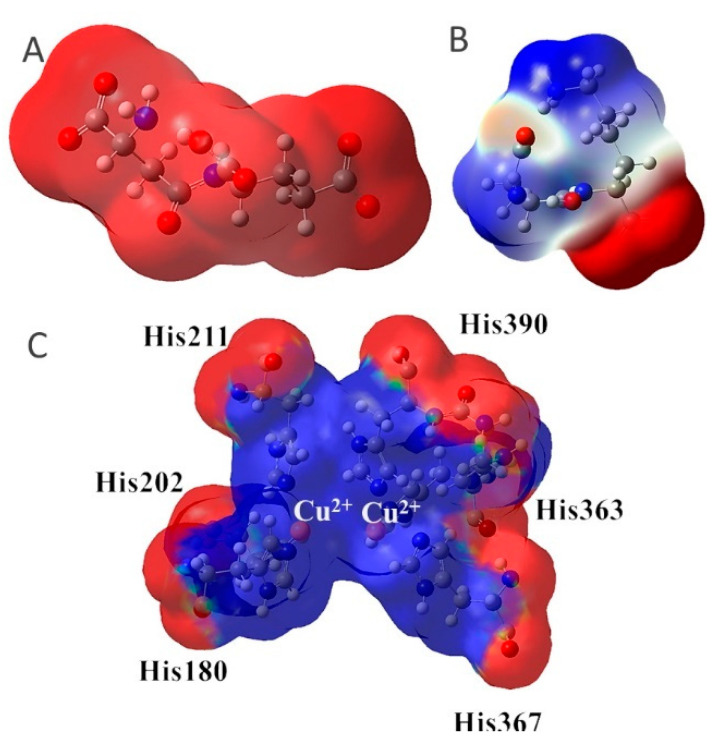
Maps of electrostatic potential for (**A**) Lig2, (**B**) Lig4, and (**C**) the active site of TYR.

The major structural difference between Lig2 and Lig4 is the presence of the amino group from lysine. Since amino groups are protonated at physiological conditions, the overall charge of the molecule for Lig4 is zero, while for Lig2 it has an overall charge of -2 (Figure 4). The electrostatic potential of the active site of TYR is positive; hence, it should more strongly attract these ligands with low ESP. However, for Lig3, which has an overall charge of −1, the binding affinities with all studied enzymes are similar to those of the other ligands.

It is also worth mentioning that the inhibition will not necessarily occur in the same way for the three enzymes although both ligands interact with TYR and TYRP1 and TYRP2 with similar binding affinities. The presence of copper atoms in the catalytic site of TYR makes it a redox enzyme, while the same is not true for TYRP1 and TYRP2 [13]. 

Moreover, one of the biggest challenges related to the design of new inhibitors based on the structure of tyrosine is due to the fact that tyrosinases can act on a wide variety of phenolic substrates. Inhibitors such as CA and related compounds can also act as substrates for the enzyme. So far, no adequate analytical tools are available to identify if the designed inhibitors are in fact substrates, i.e., are reducing the efficiency of the melanin production because they act as competitive substrates, but not as real inhibitors [8]. The advantage of the peptides in comparison to CA and tyrosine-based inhibitors relies on the absence of phenolic groups that could serve as substrates. Furthermore, the presence of electron-rich atoms such as nitrogen and oxygen in the peptides favors the chelation of copper and zinc cations [35]. Since charge interactions are stronger than Van der Waals interactions, we estimate that the peptides would bind the enzymes more efficiently than the native substrate. 

Hsiao and collaborators recently reported a series of natural and synthetic polypeptides as tyrosinase inhibitors [62]. Beyond the polypeptides, the authors also evaluated the inhibitory effects of a matrix of 20 dipeptides. Among these dipeptides, the cysteine-glutamic acid dipeptide showed the lowest IC_50_ values (IC_50_ = 2.0 μM) and is considered the most active compound. In this study, Lig2 is a dipeptide of aspartic acid and glutamic acid which showed the lowest binding affinities with the tyrosinase enzymes according to theoretical calculations. These results agree with experimental data reported in the literature. In addition, the docking studies performed by Hsiao and colleagues with mushroom tyrosinase also point out contacts through π–anion, electrostatic, and hydrogen-bonding interactions with the active site of the enzyme, which is also in agreement with the results obtained in our study.

In this study, KA was used as a model inhibitor for comparison reasons. KA is regarded as a weak inhibitor of TYR [63]. Lig2 has a comparable binding affinity to KA and could be regarded as a weak one. However, Lig2 has a significant advantage over many inhibitors—it is a nontoxic compound. Naturally derived dipeptides were reported before applications in medicine and nutrition were considered [32]. We propose further studies on Lig2 and its modifications towards higher binding affinity to TYR.

## 3. Materials and Methods

### 3.1. Protein System Setup

The in silico study of human tyrosinase-like enzymes is a challenging task, because only the tyrosinase-related protein 1 (TYRP1) structure is stored in PDB with ID 5M8L [13]. Accompanying human tyrosinase (TYR) and human tyrosinase-related protein 2 (TYRP2) were folded by AlphaFold v2.0 and updated recently in 2021 [47]. The structure of TYRP1 was taken from PDB with ID 5M8L; protein backbone and side-chain atoms were checked with ChimeraX (version: 0.93, University of California, San Francisco, CA, USA) [64] and Discovery Studio (version 21.1.0.20298, Dassault Systèmes, Paris, France). The tLeaP module of the Amber program was used to add hydrogen atoms [65]. Additionally, the structure of mushroom *A. bisporus* tyrosinase with PDB ID 2Y9X [66] was taken from PDB and subjected to the same treatment. Structures of human tyrosinase (TYR) and human tyrosinase-related protein 2 (TYRP2) were taken from the AlphaFold Protein Structure Database. In both cases, crucial cofactors for structural stability and reactivity are missing. The binding site of TYR is made of seven histidine residues, namely His180, His202, His211, His363, His367, His389, and His390, and requires two copper cations. The structure of *A. bisporus* tyrosinase (abTYR) was crystallized with two copper cations stabilized by His61, His85, His94, His259, His263, and His296. The human TYR structure obtained from AlphaFold lacks metal ions. We initially cut out the histidines that build the TYR active center and the histidines that build the abTYR active center. We found that when the two sets of histidine residues were superimposed, their geometries overlapped perfectly. We used this information and the information about the position in space of copper cations in the abTYR structure to introduce such cations into the active center of the human TYR structure. We proceeded analogously to fill in the missing zinc atoms in the active center of TYRP2. The TYRP2 active site is also composed of six histidine residues: His189, His211, His220, His369, His373, and His396.

### 3.2. Ligand Preparation 

All studied dipeptides, namely [26] aspartic acid–β-methyl ester derivative (Lig1), aspartate–glutamate (Lig2), aspartate–citrulline (Lig3), aspartate–lysine (Lig4), aspartate–arginine (Lig5), aspartate–canavanine (Lig6), aspartate–ornithine (Lig7), and aspartate–homoarginine (Lig8), as well reference ligands kojic acid (KA) and 4-hydroxy-cinnamic acid (CA), presented in Table 1, were optimized with Gaussian09 (ver. D.01, Gaussian, INC., Wallingford, CT, USA) [67] at B3LYP [68]/6-31+g(d,p) [69] level of theory. Optimized ligand structures were used to generate parameters by using GAFF [70] delivered in tLeaP (AmberTools, version 20, University of California, San Francisco, CA, USA). For each ligand, the three-dimensional plots of molecular electrostatic potential (ESP) [71] were generated; ESP measures interaction energy between the charge distribution of a molecule and a positive charge, where a negative value corresponds to an attractive interaction and a positive value corresponds to repulsion.

### 3.3. Docking Setup 

Natural tyrosinase inhibitors cinnamic acid (CA) and kojic acid (KA) and also eight dipeptide ligands were docked to abTYR, TYR, TYRP1, and TYRP2 by using the Genetic Algorithms with Unrestricted Descriptors for Intuitive Molecular Modeling (GaudiMM [72]) program v0.08The GaudiMM software was chosen because of its wide possibility to set up initial parameters and good performance for nonstandard docking tasks. Moreover, the GaudiMM code can reproduce Vina scores, which are the most widely used scores, probably due to their ease of use and speed, when compared to the other docking scores [73]. The preprocessing step was performed with default settings; all water molecules and ions, excluding cations in the active site center, were removed. The docking step was carried out by using generations equal to 200 and populations equal to 100, with the definition of the Cartesian coordinates of the binding center of the active site, the definition of six histidine residues that build the active site of enzymes, and precision parameters equal to 3. Free movements of ligands within 8 Å from the center of mass were added. During docking, the minimization of clashes to discard nonfeasible torsions was used. The HBonds objective and Vina0 score computations were performed. The resulting solutions proposed 100 ligand–enzyme complexes for each trial. The three best poses for each ligand were kept, considering clashes, Vina0 score, RMSD, and total energy. Then, one best pose of ligand in the active site was selected by key interaction analysis and subjected to binding affinity computations. 

Binding affinities. The free energy of binding (ΔG_bind_) in terms of Gibbs free energy can be described as follows:

ΔG_bind_ = G_bound_ − G_free_ = RT ln(K)

where G_bound_ is the energy of the bound complex, G_free_ defines protein and ligand separate structures, R is a gas constant, T is a temperature, and K is an equilibrium constant. Computational methods to achieve binding free energy differ in the accuracy that usually depends on computational time. The time-consuming free energy perturbation method or thermodynamics integration delivers results that may be reproduced by experimental measurements; however, their computational cost extremely limits their usage. Significantly faster are methods that use empirical functions, force-field-based potentials, statistical potentials, or scoring functions, but these models often fail for systematic prediction for large datasets or cannot discriminate between binders and nonbinders [74,75]. The conformational changes upon binding, allosteric regulation, and solvent or cofactor effects are neglected by these fast predictions. Notwithstanding, for a description of protein–ligand interactions, another approach was applied where the binding affinities were obtained by defining contact regions in the studied complex by using the network of contacts between surface residues, atoms, as it had already been used in docking studies [76,77]. Herein, the following contact region approach implemented in the Protein Binding Energy Prediction program (PRODIGY) [78] was used to compute binding affinities obtained after docking enzyme–ligand complexes.

### 3.4. Bioinformatic Analysis of Proteins

All studied protein sequences were retrieved from UniProt (TYR (P14679), TYRP1 (P17643), TYRP2 (P40126), and abTYR (C7FF44)) and trimmed to correlate to their corresponding analyzed structures. Multiple sequence alignments were performed with Clustal Omega and CLC Sequence Viewer (version 8, Qiagen, Aarhus, Denmark); domain and pattern search was completed with Pfam [79] and Prosite [80]. Two-dimensional diagrams to visualize interactions between ligands and proteins were prepared in DiscoveryStudio PyMol (version 2.5.2, Schrödinger, New York, USA).was used for protein visualization and structural analysis [].

## 4. Conclusions

Tyrosinase is the key enzyme for the biosynthesis of melanin. Due to the missing crystal structure of human tyrosinase, abTYR was largely employed as a model to find new tyrosinase inhibitors. However, the recent advances in merging artificial intelligence with life sciences succeeded in making available an outstanding-quality model of human tyrosinase structure. In this theoretical study, we employed the AlphaFold TYR structure for the first time. Similarly, the TYRP2 structure was also obtained from the AlphaFold database and used for the first time in a molecular docking project. 

Comparative studies between the abTYR crystal structure and TYR showed that although the catalytic sites of both enzymes share some similarities, the overall structures do not overlap. Moreover, when comparing the results for KA and CA for both enzymes, several differences in the interactions with the active site were found. These findings are in agreement with experimental data reported in the literature [8].

We also evaluated the interactions of KA and CA with TYRP1 and TYRP2. The binding affinities for KA and CA with these enzymes were similar to those encountered for TYR and abTYR. Since TYRP2 is an important regulator in melanin synthesis despite its dual nature and yet not very well defined activity, this enzyme is often used as a target in melanoma therapy. A better understanding of specific interactions with this enzyme is of great interest for the rational development of new potential inhibitors.

Due to the availability of such structures and through well-established methodologies of computer-aided drug design, a new library of cyanophycin-derived compounds were evaluated as potential inhibitors of the tyrosinase enzymes. Lig2 (aspartic acid–glutamate) showed the lowest binding affinities with all enzymes evaluated, except for TYRP1. Considering these results, Lig2 was identified as a leading compound. Further steps of this research would include the suggestion of a new set of molecules based on rational modifications of Lig2, as well as more advanced theoretical calculations based on the tyrosinase structures obtained from AlphaFold.

## Figures and Tables

**Table 1:** The binding affinity values obtained for selected ligands in complex with tyrosinase enzymes reported in kcal∙mol^−1^.

Ligand	abTYR	TYR	TYRP1	TYRP2
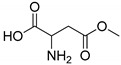 Lig1	−5.9	−6.0	−5.9	−6.0
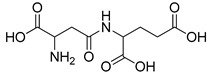 Lig2	−6.4	−6.7	−6.3	−6.8
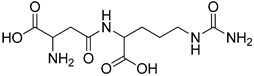 Lig3	−5.4	−5.5	−6.1	−5.7
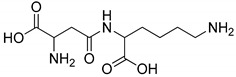 Lig4	−6.3	−6.3	−6.4	−6.6
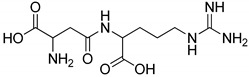 Lig5	−5.4	−5.3	−5.2	−6.0
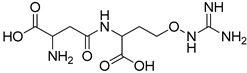 Lig6	−5.2	−5.3	−4.9	−5.6
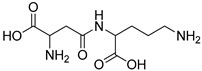 Lig7	−5.7	−5.8	−5.7	−6.1
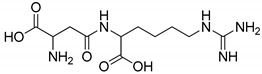 Lig8	−5.4	−5.6	−5.2	−5.4
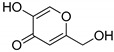 Kojic acid	−7.1	−7.3	−7.0	−7.0
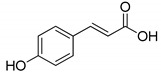 Cinnamic acid	−8.0	−8.0	−7.5	−7.8

## Data Availability

Not applicable.

## Supplementary Materials

The following supporting information can be downloaded at: https://www.mdpi.com/article/10.3390/ijms23063335/s1.
