# Supplementary material for: Theoretical Studies of Cyanophycin Dipeptides as Inhibitors of Tyrosinases
Source: Int J Mol Sci. 2022 Mar 19;23(6):3335. doi: 10.3390/ijms23063335 (PMC8950311; doi:10.3390/ijms23063335)
Supplement: Supplementary file 1 [file ijms-23-03335-s001.zip › ijms-1625532-supplementary.pdf]

International Journal of *Molecular Sciences*

MDPI

Supplementary Information

Article

Theoretical Studies of Cyanophycin Dipeptides as Inhibitors of Tyrosinases

Agnieszka Krzemińska †, Natalia Kwiatos *,†, Franciela Arenhart Soares † and Alexander Steinbüchel

International Center for Research on Innovative Biobased Materials (ICRI-BioM)—International Research Agenda, Lodz University of Technology, Żeromskiego 116, 90-924 Lodz, Poland; agnieszka.krzeminska-kowalska@p.lodz.pl (A.K.); franciela.arenhart-soares@p.lodz.pl (F.A.S.); alexander.steinbuchel@p.lodz.pl (A.S.)

* Correspondence: natalia.kwiatos@p.lodz.pl

† These authors contributed equally to this work

Citation: Krzemińska, A.; Kwiatos, N.; Arenhart Soares, F.; Steinbüchel, A. Theoretical Studies of Cyanophycin Dipeptides as Inhibitors of Tyrosinases. *Int. J. Mol. Sci.* **2022**, *23*, 3335. https://doi.org/10.3390/ijms23063335

Academic Editor: Vitaliy Borisov

Received: 19 February 2022
Accepted: 17 March 2022
Published: 19 March 2022

Publisher's Note: MDPI stays neutral with regard to jurisdictional claims in published maps and institutional affiliations.

Copyright: © 2022 by the authors. Licensee MDPI, Basel, Switzerland. This article is an open access article distributed under the terms and conditions of the Creative Commons Attribution (CC BY) license (https://creativecommons.org/licenses/by/4.0/).

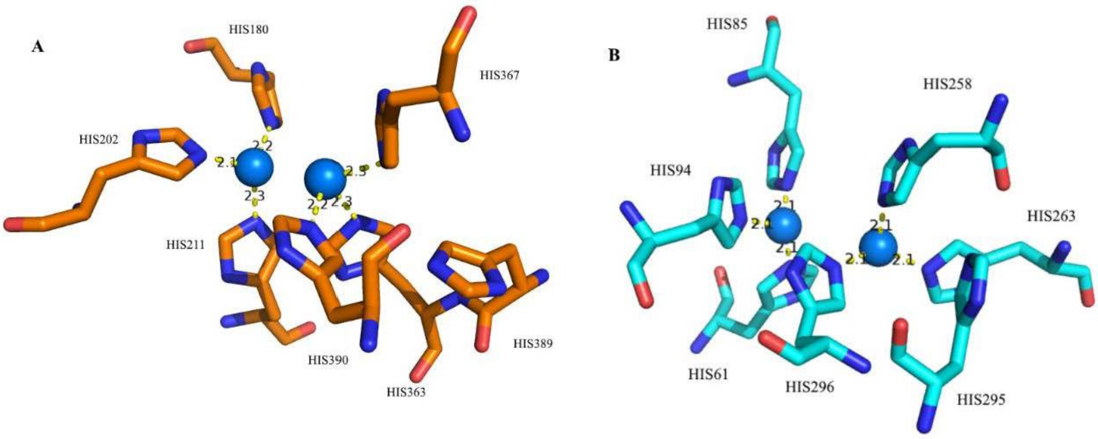

Figure S1. The distances between copper atoms at (**A**) TYR and (**B**) abTYR.

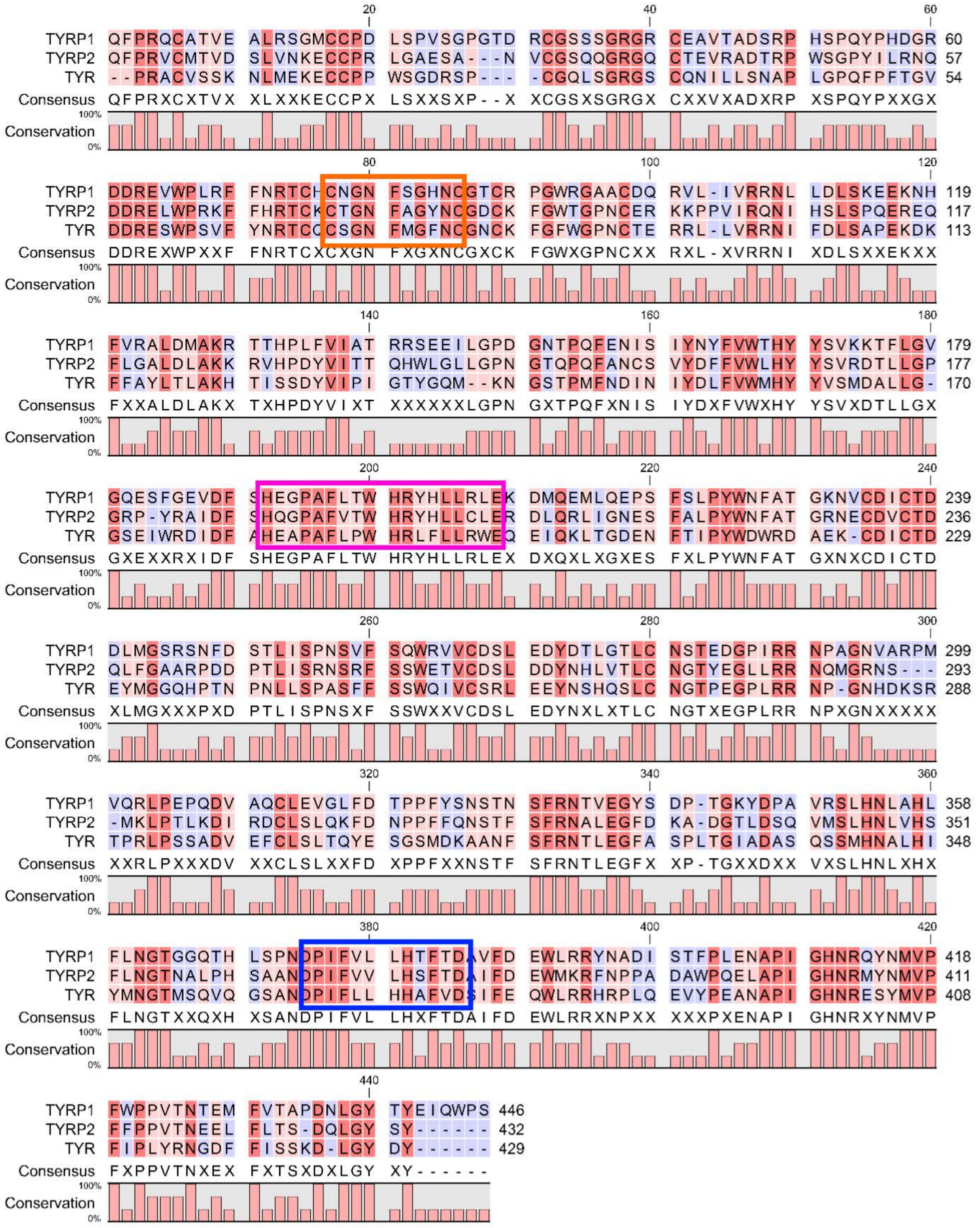

Figure S2. Multiple Sequence Alignment of TYR, TYRP1 and TYRP2. Magenta—CuA binding pattern, blue—CuB binding pattern, orange EFG pattern.

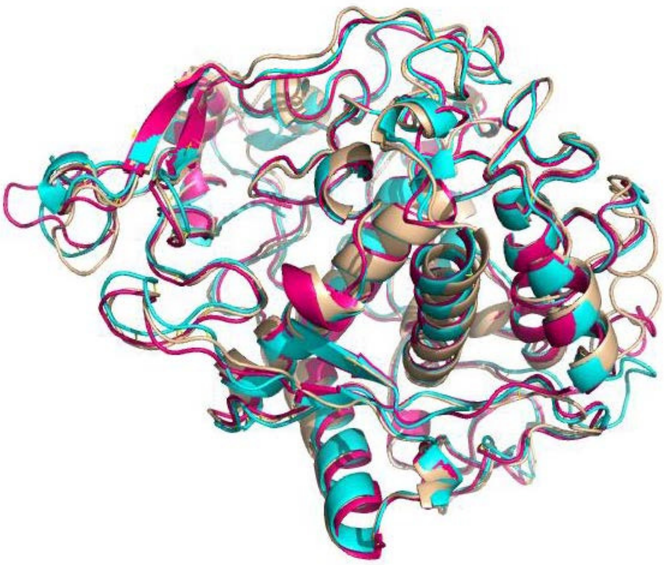

Figure S3. Alignment of TYR (wheat), TYRP1(magenta) and TYRP2 (cyan).

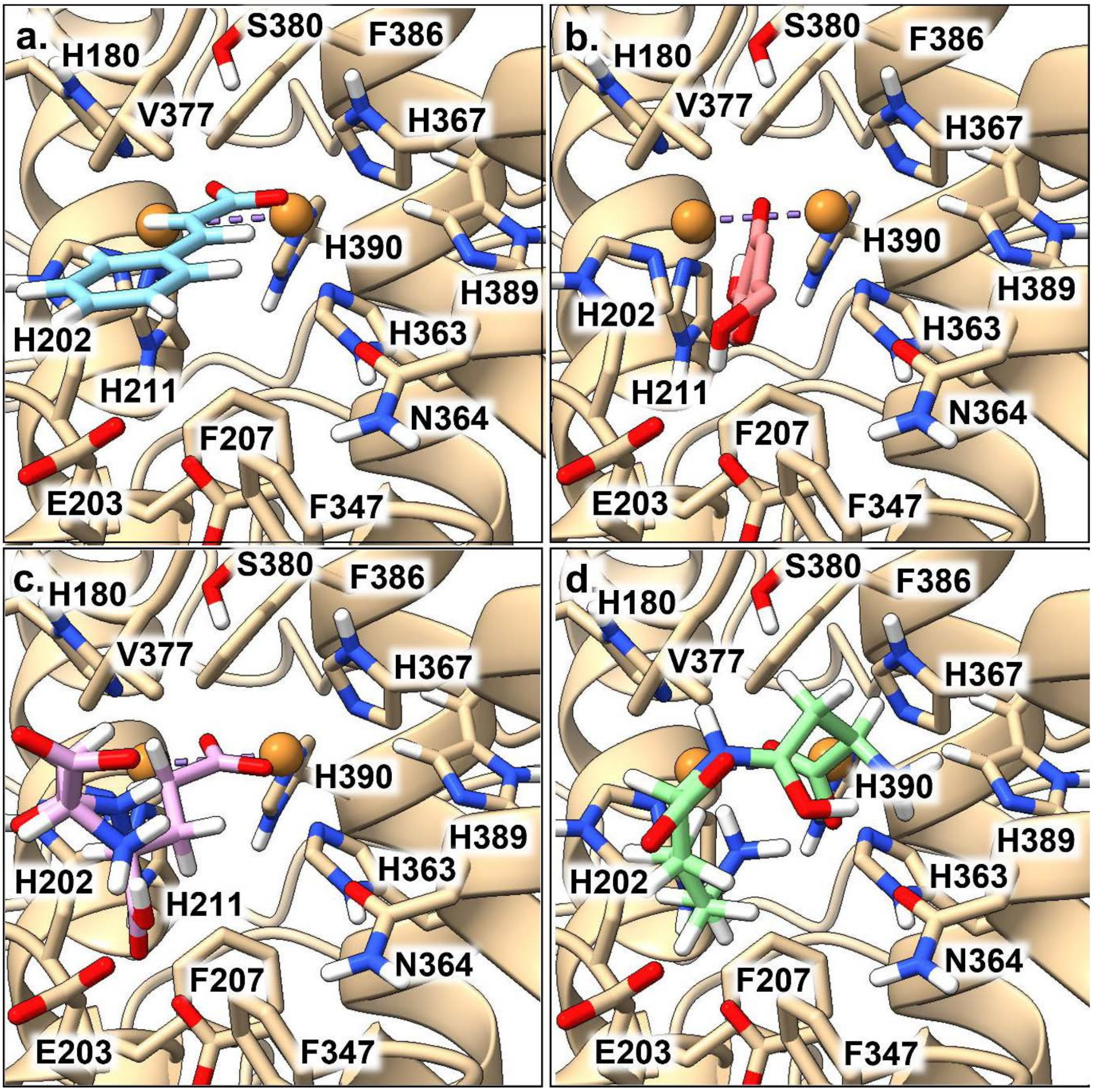

Figure S4. Ribbon representation of the TYR active site with two copper atoms (orange balls) in complex with (**a**) cinnamic acid, (**b**) kojic acid, (**c**) aspartate-glutamate (lig2), and (**d**) aspartate-lysine (lig4). Key amino acids that create interactions with ligands are labeled in black.

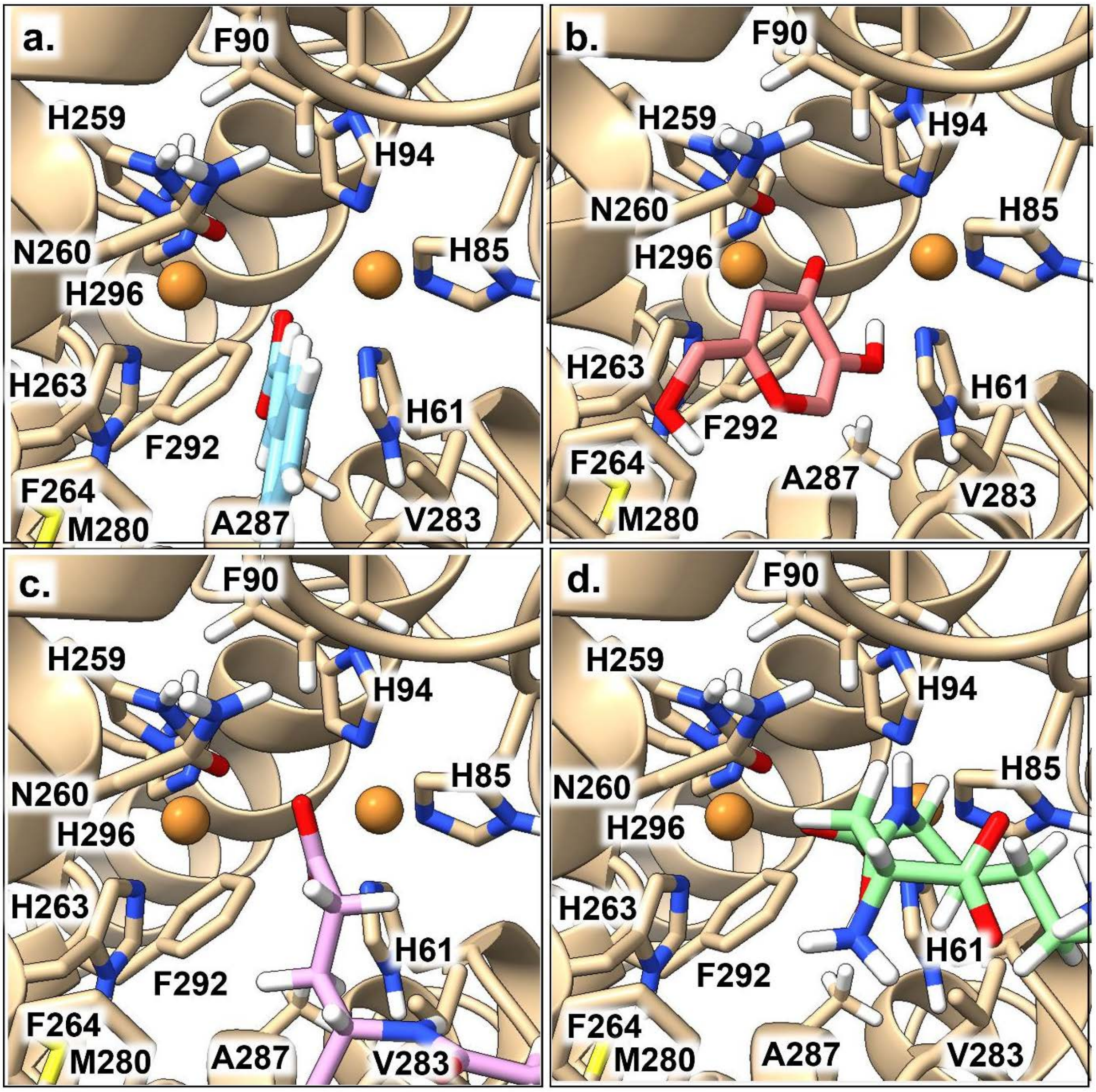

Figure S5. Ribbon representation of the abTYR active site with two copper atoms (orange balls) in complex with (**a**) cinnamic acid, (**b**) kojic acid, (**c**) aspartate-glutamate (lig2), and (**d**) aspartate-lysine (lig4). Key amino acids that create interactions with ligands are labeled in black.

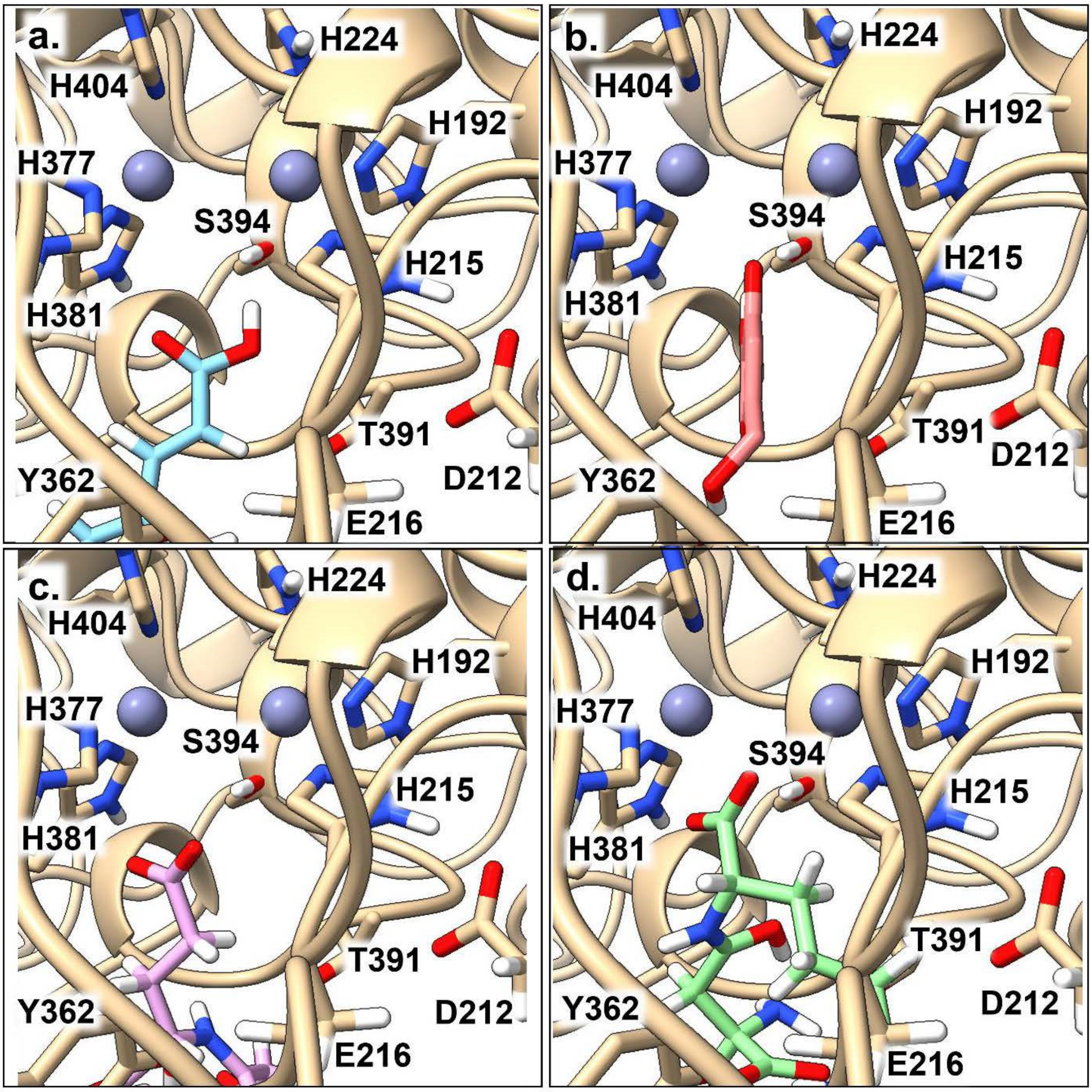

Figure S6. Ribbon representation of the TYRP1 active site with two zinc atoms (blue balls) in complex with (**a**) cinnamic acid, (**b**) kojic acid, (**c**) aspartate-glutamate (lig2), and (**d**) aspartate-lysine (lig4). Key amino acids that create interactions with ligands are labeled in black.

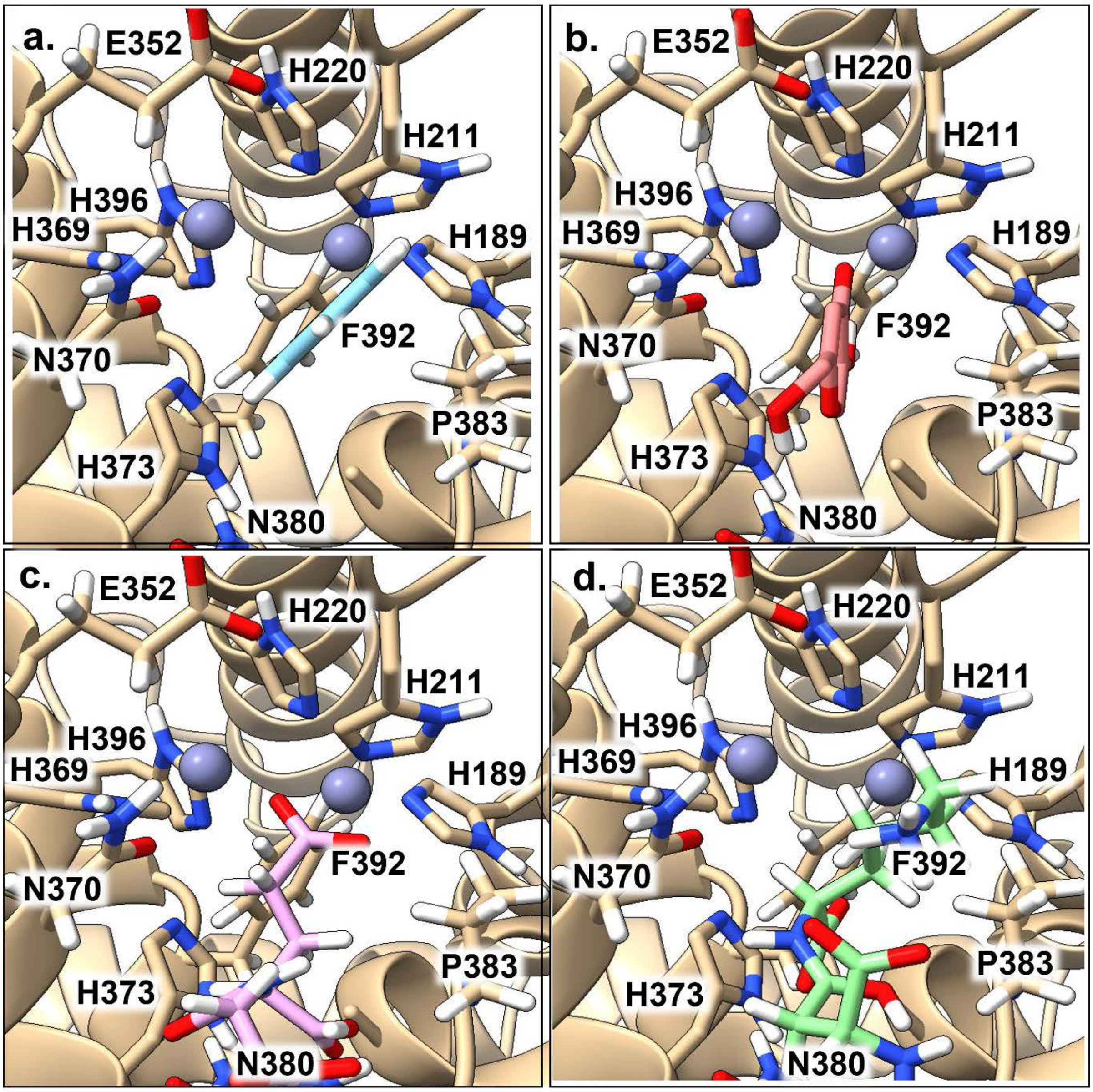

Figure S7. Ribbon representation of the TYRP2 active site with two zinc atoms (blue balls) in complex with (**a**) cinnamic acid, (**b**) kojic acid, (**c**) aspartate-glutamate (lig2), and (**d**) aspartate-lysine (lig4). Key amino acids that create interactions with ligands are labeled in black.
